# Supplementary figures and images for: Seroprevalence of immunoglobulin G antibodies against SARS-CoV-2 in Cyprus
Source: PLoS One. 2022 Jun 13;17(6):e0269885. doi: 10.1371/journal.pone.0269885 (PMC9191710; doi:10.1371/journal.pone.0269885)

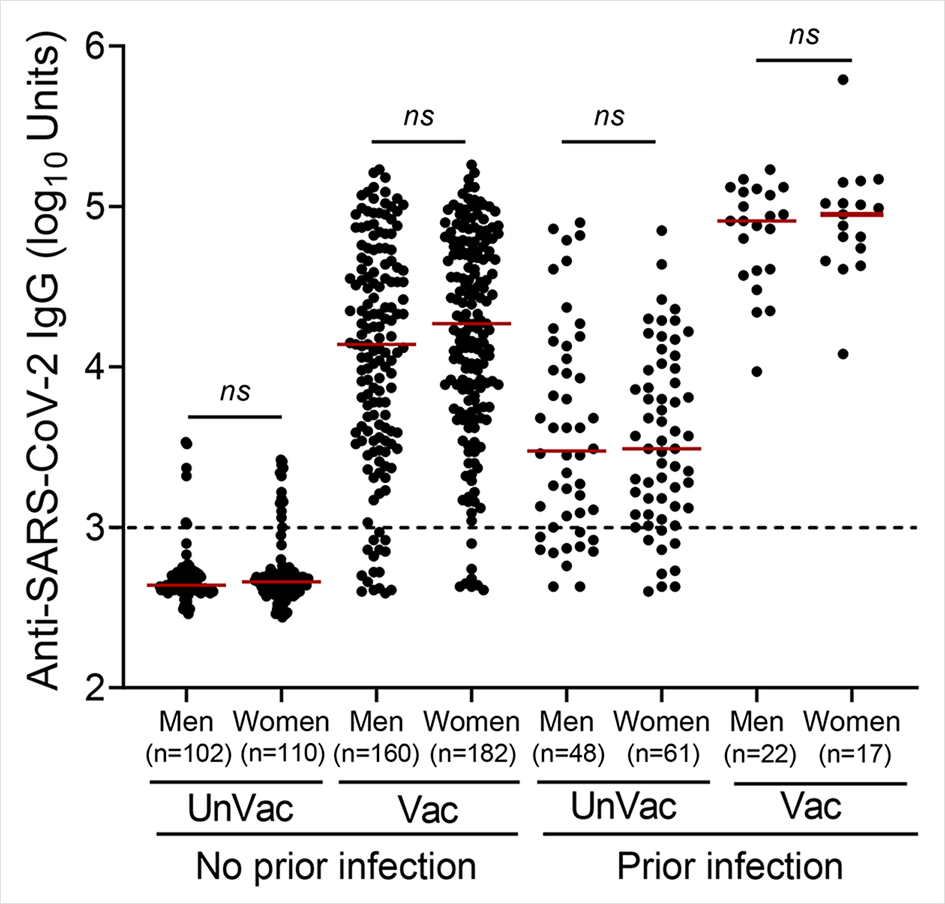

Supplement: S1 Fig — Participants were not vaccinated (UnVac) or received at least one dose of a vaccine against SARS-CoV-2 (Vac). The number of participants (n) in each group is shown in parentheses. Mann-Whitney U-test was used for pairwise statistical analysis. There were no significant (ns) differences in anti-spike IgG antibody responses between men and women in all groups. Horizontal bars indicate median values. Dotted line: positive cut-off value. (TIF) [file pone.0269885.s001.tif]

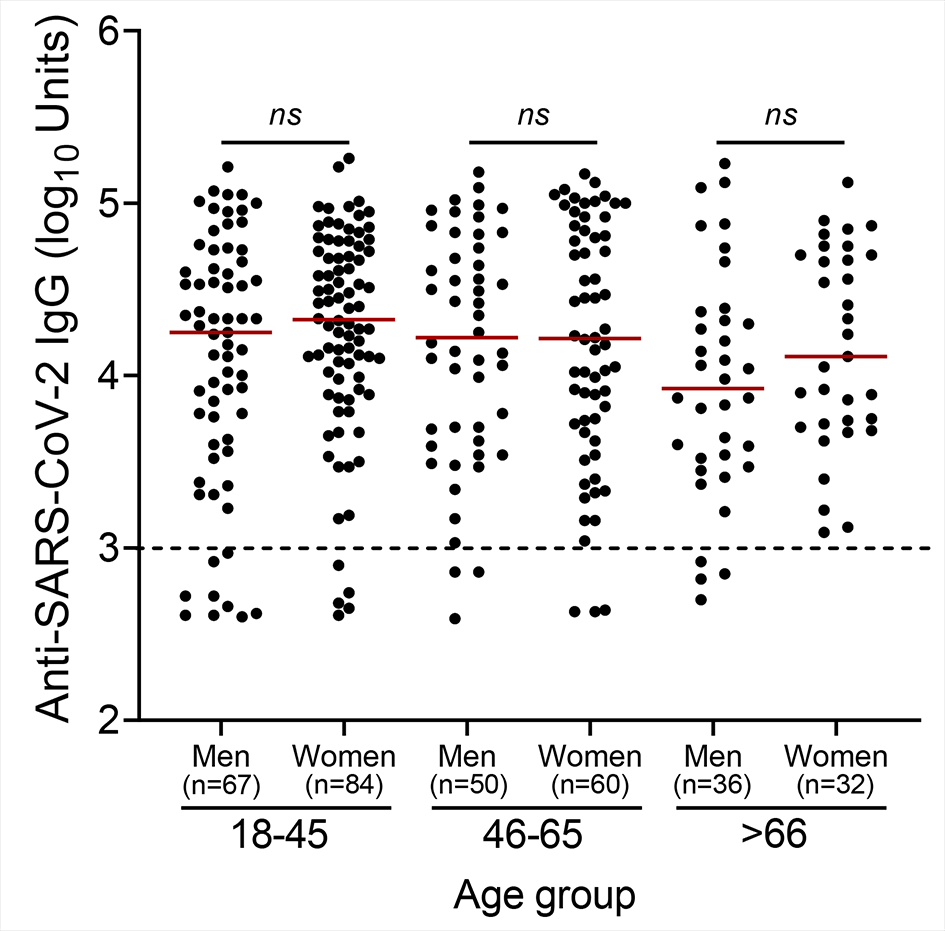

Supplement: S2 Fig — The number of participants (n) in each group is shown in parentheses. Mann-Whitney U-test was used for pairwise statistical analysis. There were no significant (ns) differences in anti-spike IgG antibody responses between men and women in all groups. Horizontal bars indicate median values. Dotted line: positive cut-off value. (TIF) [file pone.0269885.s002.tif]

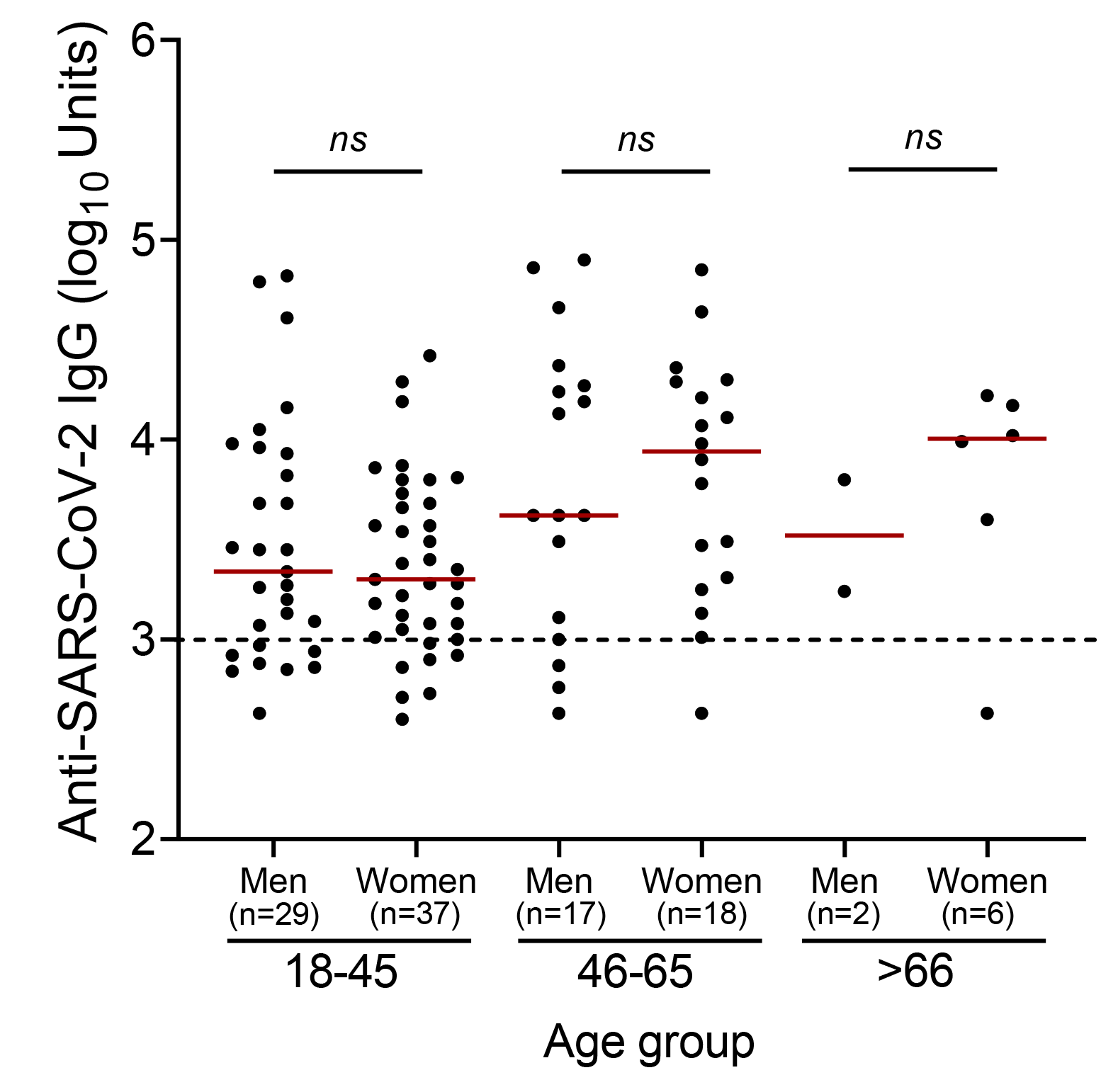

Supplement: S3 Fig — All participants were not vaccinated against SARS-CoV-2. The number of participants (n) in each group is shown in parentheses. Mann-Whitney U-test was used for pairwise statistical analysis. There were no significant (ns) differences in anti-spike IgG antibody responses between men and women in all groups. Horizontal bars indicate median values. Dotted line: positive cut-off value. (TIF) [file pone.0269885.s003.tif]
