# Supplementary material for: Seroprevalence of immunoglobulin G antibodies against SARS-CoV-2 in Cyprus
Source: PLoS One. 2022 Jun 13;17(6):e0269885. doi: 10.1371/journal.pone.0269885 (PMC9191710; doi:10.1371/journal.pone.0269885)
Supplement: Attachment — Submitted filename: Comments_PONE-D-22-04892_reviewer_04.04.22.pdf [file pone.0269885.s004.pdf]

Comments to the author:

In this manuscript, the authors used blood samples from 702 participants from four cities in Cyprus (Europe) to estimate the seroprevalence of SARS-CoV2 IgG antibody and to compare the antibody levels among three groups of participants including: i) vaccinated without evidence of the previous infection, ii) unvaccinated with evidence of the previous infection, and iii) vaccinated with evidence of the previous infection. The results from this study revealed that the highest response of virus-specific IgG antibodies were observed in individuals who were infected with SARS-CoV-2 and received at least one dose of a vaccine. The results also indicate that vaccine-induced responses lead to higher IgG antibodies response compared to those produced following infection with the virus.

I find the study noteworthy because 1) the data overall for SARS-CoV2 infection in Cyprus is limited, and 2) the study employed a large sample size and different groups which strengthens the study. Here are several points that need to be considered:

Major points:

- I would suggest analyzing some of the SARS-2 IgG positive samples in the viral neutralization assay to determine the magnitude of neutralizing antibodies (NAbs).
- The manuscript lack samples from different time points. Therefore, I would suggest the authors include flow-up samples if that is possible for the durability of the IgG responses.

Minor points:

- The abstract section is poorly written. Therefore I would suggest the authors improve the abstract by summarizing/revising the current abstract.
- What are the methods used to determine the previous SARS-2. I encourage the author to state that in the methos section.
- Since the blood samples were collected in the anticoagulant tube, samples should be plasma not serum samples, I encourage the authors correct that in the method section.
- P6 and P7: in the methods section, subtitle laboratory testing, I suggest the authors to describe the assays in this section separately, for instance, ELISA should be in the separate subtitle with more details..……..etc.
- The specificity and sensitivity of the ELISA assay should be mentioned in the method section.
- Since the collected samples from participants visited the clinic for routine check-up or other tests, Are the patients have SARS-CoV2 acute symptoms? If so, are the samples tested for the IgM antibody? It would be nice if the authors include the IgM data since some samples maybe collected during the acute phase of infection.

- The authors should describe how they determine the antibody level somewhere in the manuscript. otherwise, the term IgG level should be changed to the IgG titer if they already checked the titer if not they should write IgG response.
- The IgG subclass need to be tested at least for the samples that show high IgG response.
- The study inclusion/exclusion criteria in general need further explanation for the reader. For example, were subjects with COVID-19 symptoms excluded? What tests were performed to exclude other common causes of COVID-19 disease-like symptoms?
- P20L456-457: The authors stated that their results also “suggest that vaccine-induced responses are significantly more effective than natural immunity alone” I think this statement needs to be discussed /explained in more detail in the discussion section.
- The discussion should describe the findings in a more concise manner.

In general, this work deals with an important topic and provides useful information to the reader. However, given all the above concerns, I believe this manuscript requires fixing the points before being accepted for publication.
