# Supplementary material for: Seroprevalence of immunoglobulin G antibodies against SARS-CoV-2 in Cyprus
Source: PLoS One. 2022 Jun 13;17(6):e0269885. doi: 10.1371/journal.pone.0269885 (PMC9191710; doi:10.1371/journal.pone.0269885)
Supplement: Attachment — Submitted filename: PONE-D-22-04892R1.pdf [file pone.0269885.s006.pdf]

Comments to the author:

This work deals with an important topic and provides useful information to the reader. I believe the manuscript has been improved a lot.

Minor points:

- Some areas of the manuscript require significant proofreading and revision to improve the quality of English for instance the abstract section has unclear sentences.
- Source of the regents should be provided in the method section (antibody measurement section).
