# Supplementary material for: Seroprevalence of immunoglobulin G antibodies against SARS-CoV-2 in Cyprus
Source: PLoS One. 2022 Jun 13;17(6):e0269885. doi: 10.1371/journal.pone.0269885 (PMC9191710; doi:10.1371/journal.pone.0269885)
Supplement: Attachment — Submitted filename: PONE-D-22-04892R1_response to Reviewers comments 180522.docx [file pone.0269885.s007.docx]

**[PONE-D-22-04892R1] - [EMID:466ddddc0f05a1c8]**

**Reviewer #1:**

Comments to the author:

This work deals with an important topic and provides useful information to the reader. I believe the manuscript has been improved a lot.

Minor points:

- Some areas of the manuscript require significant proofreading and revision is needed to improve the quality of English for instance the abstract section has unclear sentences.

The manuscript was reviewed and revised accordingly.

- Source of the reagents should be provided in the method section (antibody measurement section).

The source of reagents is provided in the revised manuscript (Siemens Healthcare Diagnostics Inc., Erlangen, Germany).

**Reviewer #2:**

The authors have partially addressed my concerns. Here are the remaining concerns to be addressed:

1. Line 170: Please include the resource (company) where the fluoresceinated S1 receptor-binding RBD antigen was purchased, including the catalog #. If the antigen was made in house, please include the sequence information.

The following text was added to the manuscript to address reviewer’s comment

“*All components for the detection of anti-IgG against SARS-CoV-2 including the fluoresceinated S1 receptor-binding RBD antigen were included in the Dimension Vista SARS‑CoV‑2 IgG (COV2G) assay (Siemens Healthcare Diagnostics Inc., Erlangen, Germany, Cat #K7771
11417771)*”

2. Line 29: please add “,” after this study, and change “plasma the” to “the plasma”.

Corrected

3. Line 42: please change “humoral immunity” to “antibody responses”

Corrected
